# Lack of maternal exposure to somatostatin leads to diet-induced insulin and leptin resistance in mouse male offspring

**DOI:** 10.1530/JME-24-0102

**Published:** 2025-03-22

**Authors:** Zhongyue Yang, Catherine P Kirschke, Liping Huang

**Affiliations:** ^1^ Department of Nutrition, University of California at Davis, Davis, California, USA; ^2^ USDA/ARS/Western Human Nutrition Research Center, Davis, California, USA; ^3^ Integrative Genetics and Genomics, University of California at Davis, Davis, California, USA

**Keywords:** somatostatin, diet-induced obesity, glucose intolerance, insulin resistance, maternal exposure, leptin resistance

## Abstract

Somatostatin (Sst) is an inhibitory regulator of many hormones. The prenatal environment impacts an offspring’s risk to type 2 diabetes in adulthood. However, the effect of maternal *Sst* deficiency on glucose and insulin metabolism in offspring and metabolic disease risk in their adult life has been poorly understood. The study was to investigate the impact of a lack of maternal *Sst* exposure in mouse male and female offspring on diet-induced changes in glucose metabolism and adiposity. *Sst* knockout offspring, *Sst*KO born to the *Sst*-heterozygous dams or *Sst*KO-MSD born to the *Sst*-homozygous dams were fed either a regular diet (CD) or a high-fat diet (HFD) at 3-week-old for 15 weeks. Body weight and blood glucose levels were monitored. Glucose and insulin tolerance tests were performed. Plasma hormone levels and gene expression in the hypothalamus were investigated. The results demonstrated that only male *Sst*KO-MSD offspring developed obesity accompanied by severe insulin and leptin resistance after HFD challenge. Insulin secretion was reduced in both basal and oral glucose-challenged conditions in the CD-fed male *Sst*KO-MSD mice. A reduced ratio of islet area to pancreas area was noted in *Sst*KO-MSD mice in both sexes. Plasma levels of glucagon, Glp1 and Pyy were elevated in both male and female *Sst*KO and *Sst*KO-MSD mice. mRNA expression of *leptin receptor*, *FoxO1*, *Npy* and *Agrp* was downregulated in male *Sst*KO-MSD mice. These results demonstrate that a lack of fetal somatostatin exposure impairs the islet development in offspring and increases risk of obesity, insulin resistance and leptin resistance later in life.

## Introduction

A principle in the field known as the ‘Developmental Origins of Health and Disease’ (DOHaD) postulates that adverse intrauterine environmental exposure in early life, especially during gestation, can escalate the prevalence of chronic metabolic disorders in offspring. This includes diabetes, obesity and dyslipidemia, which can persist in adulthood and even across generations (Gluckman *et al.* 2010, Dunkerton & Aiken 2022, Wu *et al.* 2023). Several factors contribute to the intrauterine environment, including maternal nutritional status (Alejandro *et al.* 2014), maternal immune challenge (Nilsson *et al.* 2001), physical activity (Laker *et al.* 2014), psychological stress (Balasubramanian *et al.* 2015) and tobacco utilization during gestation (Rogers 2019). Most importantly, the genetic background of the mother has the most direct impact on the intrauterine environment (Burchat *et al.* 2021), which includes genetic constitution, DNA methylation, histone modifications, noncoding RNAs and miRNA.

In addition to the maternal intrauterine environment, an after-weaning diet plays a profound role in affecting the risk of chronic metabolic diseases in offspring. Multiple rodent models have demonstrated the importance of maternal environment and after-weaning diets on the offspring’s health in later life. For example, one study using a genetically obese mouse model (Ay/a) demonstrated that transgenic dams carrying and transmitting the human 8-oxoguanine DNA glycosylase gene (*OGG1*) protected transgenic offspring against diet-induced obesity through increased mitochondrial contents in adipocytes that balanced energy metabolism (Burchat *et al.* 2021). In addition, Levin *et al.* reported that offspring from Sprague–Dawley rat dams predisposed to obesity (DIO) were more sensitive to diet-induced obesity with elevated leptin and plasma glucose levels than those from obese-resistant dams (DR) when the dams were challenged with a high-fat diet (HFD) (Levin *et al.* 1997, Levin & Govek 1998). The obese-prone or resistant phenotype in the offspring was found to be caused by both the maternal genotype (DIO or DR) and the intrauterine environment (obese dams or not) via altered sensitivity of the offspring’s ventromedial nucleus neurons to glucose and long-chain fatty acids (Le Foll *et al.* 2009) and reduced leptin sensitivity (Ricci & Levin 2003, Irani *et al.* 2009).

Somatostatin (human SST or mouse Sst) is a peptide hormone that is secreted from the δ-cell localized in many tissues, including the islets of the pancreas, the hypothalamus of the brain and the stomach and intestine of the gastrointestinal tract (Arimura *et al.* 1975, Reichlin 1983*a*). SST is a known neuropeptide expressed in the brain as an inhibitory hormone, specifically inhibiting the release of growth hormone (GH) in the pituitary gland (Reichlin 1983*a*). SST is also present in the δ-cell of the gut mucosa and the myenteric neural plexus. Through its paracrine and endocrine actions, SST suppresses the releases of glucagon-like peptide 1 (GLP1), glucose-dependent insulinotropic polypeptide (GIP), gastrin, vasoactive intestinal polypeptide, ghrelin and cholecystokinin (CCK) in the gastrointestinal tract (Reichlin 1983*a*,*b*). *Sst*-deficient mice exhibit increased baseline plasma GH, corticosterone and total ghrelin levels and impaired motor performance (Zeyda & Hochgeschwender 2008). *Sst*-deficient mice are also more sensitive to diet-induced metabolic abnormalities, such as increased fat accumulation and impaired glucose and insulin metabolism, especially in males (Luque *et al.* 2016*a*).

The maternal influence of SST on fetal development and health has been of great interest to scientists since the 1970s. Studies have shown that the human placenta is responsive to SST (Caron *et al.* 1997) and newborns have elevated SST levels in the circulation (Goldstein *et al.* 1995). SST is also present in the fetal pancreas from an early stage of development, as its level increases along with the gestational age (Mcintosh *et al.* 1977). However, it has been shown that human fetal β-cells appear to be relatively insensitive to SST (Otonkoski *et al.* 1993), leading to an inconclusive impact of SST on the human fetal pancreas. Nevertheless, the impact of maternal SST deficiency on offspring metabolic health later in life, especially during a HFD challenge, remains unknown. The current study investigated the effects of a lack of maternal Sst exposure on obesity development, glucose tolerance and peripheral insulin sensitivity in offspring challenged with or without a HFD. The secretion of Sst-regulated peptide hormones in the circulation was also studied along with the mRNA expression levels of genes that were affected by the Sst receptor signaling pathway in the hypothalamus of the brain. Our findings suggest that a lack of Sst exposure in the uterus may impair islet development in the pancreas in mice, resulting in predisposition to diet-induced obesity and insulin resistance.

## Materials and methods

### Mice, breeding and diets

**Figure 1:**
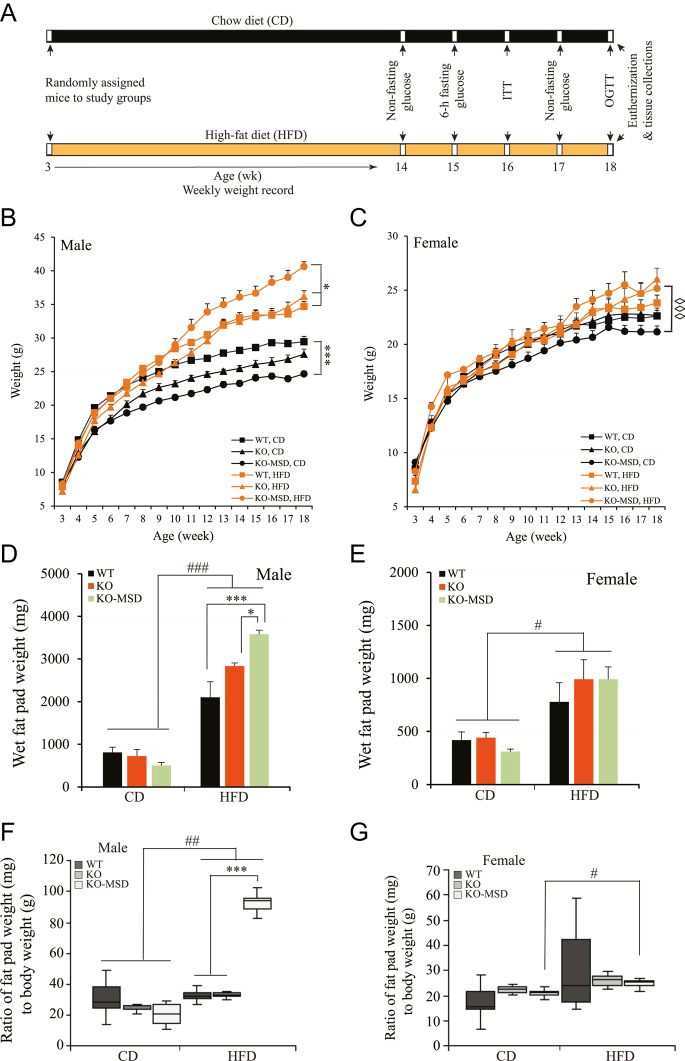
The schematic experimental design (A), growth curve of male (B) and female (C) mice fed CD or HFD, respectively, fat pad weight of male (D) and female (E) mice fed either CD or HFD, respectively, ratio of fat pad weight (mg) to body weight (g) of male (F) and female (G) mice, respectively. The data are shown as the mean ± SEM (*n* = 7–12/male group and *n* = 8–10/female group). *P < 0.05 and ***P < 0.001 indicate the significant difference between genotypic groups in the same dietary regime. ◇◇◇*P* < 0.001 indicates the significant difference between diets in the same genotypic group. ^#^*P* < 0.05 and ^###^*P* < 0.001 indicate the significant difference between diets across the genotypic groups. A one-way or two-way ANOVA test followed by a Tukey’s multiple comparisons test with adjusted *P* values was applied. WT, wildtype; KO, *Sst* knockout; KO-MSD, *Sst* knockout born to the *Sst*KO mothers; chow, regular chow diet; HFD, high-fat diet; wk, week; ITT, insulin tolerance test; OGTT, oral glucose tolerance test. A full color version of this figure available at https://doi.org/10.1530/JME-24-0102.

*Sst*-heterozygous mice (B6.N.129S4(129S6)-^*Sst*tm1Ute^/J) were cryorecovered from the Jackson Laboratories (Stock No. 008117; Bar Harbor, Maine). *Sst* knockout mice (*Sst*KO) were obtained from 18 litters produced by 14 heterozygous females mated to either heterozygous (eleven litters) or homozygous males (seven litters). The wildtype control (WT) was obtained from 14 litters by 12 heterozygous females mated to *Sst*-heterozygous males. The average litter size for the heterozygous mating was 6.9 ± 0.4 SEM. The average litter size for heterozygous females mated to homozygous males was 7.6 ± 0.7 SEM. *Sst*KO-MSD (offspring from maternal *Sst* deficiency) were generated from the *Sst*-homozygous mating (litter, *n* = 12 generated from five dams). The average litter size for the homozygous mating was 6.2 ± 0.4 SEM. No significant difference was observed in the litter size among the genotypic mating groups (*P* > 0.05). All mice were housed in a temperature-controlled room at 22–24°C with a 12-h light:12-h darkness cycle. Breeding mice were fed a standard laboratory chow diet (Laboratory Rodent Diet 5001, LabDiet, USA) and double-distilled water *ad libitum*. Mice were genotyped at 2-weeks-old, weaned at 3-weeks-old and then randomly assigned to either a chow diet (CD) group or a HFD group (45% kcal fat; D12451, Research Diets, USA) *ad libitum* for 15 weeks (Fig. 1A). Body weights were measured weekly. At 14 and 17 weeks of age, non-fasting blood glucose levels were recorded at 08:00–09:00 h using an EasyTouch® blood glucometer (MHC Medical Products, LLC, USA). At 15 weeks of age, mice were fasted from 08:00 h to 14:00 h, and then 6 h fasting glucose levels were determined. At 16 weeks of age, mice were fasted for 4 h from 08:00 h to 12:00 h and insulin tolerance tests were performed. At the end of the study (18 weeks of age), mice were fasted overnight (16–18 h) and oral glucose tolerance tests were conducted. At necropsy, whole blood was collected via cardiac puncture under general anesthesia (ketamine/xylazine at the dose of 100/10 mg/kg via an intraperitoneal injection), followed by exsanguination. Plasma was then isolated by centrifuging blood samples at 960 ***g*** at 4°C for 10 min. Epididymal or periovarian and retroperitoneal fat pads were isolated and weighed. Other tissues, including the pancreas and hypothalamus, were collected for subsequent experiments. All animal experiments were conducted in accordance with the National Institutes of Health Guidelines for the Care and Use of Experimental Animals and were approved by the Institutional Animal Care and Use Committee of the University of California, Davis.

### Genotyping

Genomic DNA was isolated from the mouse tail using a DNeasy Tissue Kit (Qiagen, USA). PCR was used for genotyping. Primer information is given in Table 1. PCR products of *Sst*Het mice yielded two distinct bands: a 1,000 bp DNA fragment (the *Sst* knockout allele) and a 469 bp DNA fragment (the WT allele), according to the protocol provided by the Jackson laboratories. PCR products were detected by agarose gel electrophoresis.

**Table 1:** Primers for genotyping the B6N.129S4(129S6)-*Sst*^tm1Ute^/J mouse strain*.

Primer name	Forward primer sequence (5′ → 3′)	Reverse primer sequence (5′ → 3′)	Genotype
*Sst*-WT	TCA​GTT​TCT​GCA​GAA​GTC​TCT​GGC	GAATGCCAATAGTTTGCGCAGCTCC	WT
*Sst*-Mu	ATC​CAG​GAA​ACC​AGC​AGC​GGC​TAT	GAATGCCAATAGTTTGCGCAGCTCC	*Sst*KO

Primer information was provided by the Jackson laboratories.

* JAX stock #008117.

### Oral glucose tolerance test

Mice were fasted overnight (16–18 h). The next morning, mice were weighed and fasting glucose levels were determined using an EasyTouch® blood glucometer. Sterilized glucose solution (20%; 0.2 μm filtered) was administered (1.5 g/kg of body weight) directly into the stomach via oral gavage. Blood was collected from the submandibular vein before (0-min) and after the glucose administration (15-, 30-, 60- and 120-min). Blood glucose levels were determined using the EasyTouch glucometer at 15-, 30-, 60- and 120-min. Plasma was purified by centrifuging blood samples at 960 ***g*** at 4°C for 10 min and stored at −80°C until use.

### Intraperitoneal insulin tolerance test

Before the test, mice were fasted for 4 h and weighed. Insulin (5.5 units of Humulin^®^ R (U-100)/kg of body weight; Eli Lilly, USA) was given intraperitoneally. Glucose levels were determined at 0-, 30-, 60- and 120-min, as mentioned above after the insulin injection via tail vein sampling.

### RNA purification, cDNA synthesis and quantitative RT-PCR

Total RNA was purified from the hypothalamus using an Aurum™ total RNA mini kit including a DNase treatment, according to the manufacturer’s instructions (Bio-Rad Laboratories, USA). RNA concentrations were determined by a NanoDrop instrument (Thermo Fisher Scientific, USA). cDNA was synthesized from 100 ng of total RNA using a high-capacity cDNA reverse transcription kit (Thermo Fisher Scientific), according to the manufacturer’s instructions. cDNA was diluted by five-fold with Ultrapure™ distilled water (Thermo Fisher Scientific). The PCR reaction mix was made in a PowerUp™ SYBR™ Green Master Mix (Applied biosystems, Thermo Fisher Scientific) containing 2 μL diluted cDNA sample, 1 μL 5 pmol/μL forward primer and 1 μL 5 pmol/μL reverse primer of the target gene (Table 2). The primer sequences were obtained from the Primer Bank (https://pga.mgh.harvard.edu/primerbank). All primers were synthesized by Life Technologies (Thermo Fisher Scientific). PCR was performed on a QuantStudio™ 7 flex real-time PCR system (Applied biosystems, Thermo Fisher Scientific). Quantitative PCR was run in triplicate and the average cycle number (Ct) was obtained. The amount of the target transcripts was then normalized to the amount of the *Eef2* transcripts by subtracting the Ct of the target from the Ct of *Eef2* (ΔCt).

**Table 2:** Primers used in the gene expression study.

Gene name	Forward primer sequence (5′ → 3′)	Reverse primer sequence (5′ → 3′)	PrimerBank ID
*Leptr*	TGG​TCC​CAG​CAG​CTA​TGG​T	ACC​CAG​AGA​AGT​TAG​CAC​TGT	28077101a1
*FoxO1*	CCC​AGG​CCG​GAG​TTT​AAC​C	GTT​GCT​CAT​AAA​GTC​GGT​GCT	34328255a1
*Ptpn1*	GGA​ACT​GGG​CGG​CTA​TTT​ACC	CAA​AAG​GGC​TGA​CAT​CTC​GGT	6755238a1
*Npy*	ATG​CTA​GGT​AAC​AAG​CGA​ATG​G	TGT​CGC​AGA​GCG​GAG​TAG​TAT	12963683a1
*Agrp*	ATG​CTG​ACT​GCA​ATG​TTG​CTG	CAG​ACT​TAG​ACC​TGG​GAA​CTC​T	6671529a1
*Pomc*	ATG​CCG​AGA​TTC​TGC​TAC​AGT	TCC​AGC​GAG​AGG​TCG​AGT​TT	6679415a1
*Socs3*	ATG​GTC​ACC​CAC​AGC​AAG​TTT	TCC​AGT​AGA​ATC​CGC​TCT​CCT	6671758a1
*Eef2*	TGT​CAG​TCA​TCG​CCC​ATG​TG	CAT​CCT​TGC​GAG​TGT​CAG​TGA	33859482a1

### Histological analysis and quantification of islet mass

The whole pancreas was dissected from experimental mice at necropsy and placed in 4% paraformaldehyde solution made in 1× PBS, pH 7.4 overnight. The pancreas was then rinsed in 1× PBS for 30 min at room temperature (RT) three times, followed by 40 min in 80% FLEX (Richard-Allan Scientific, USA) and placed at 4°C until further processing. Dehydration steps consisted of 95% FLEX (Richard-Allan Scientific, USA) for 20 min twice, 100% FLEX (Richard-Allan Scientific) 20 min twice and Clear-Rite 3 (Richard-Allan Scientific) for 15 min twice. The pancreas was then infiltrated and embedded in Type 6 paraffin (Richard-Allan Scientific) at 58°C with three rotations, and the last rotation was under vacuum pressure of 20 in/Hg for 30 min. The pancreas was sectioned in 5 μm thickness, mounted on a positive-charged slide, dried first at 42°C for 30 min and then at 58°C for 45 min. Hematoxylin and eosin (H&E) staining was performed according to a published protocol (Cardiff *et al.* 2014). Photomicrographs of the whole pancreas were acquired using an EVOS imaging system (Thermo Fisher Scientific). The area of the pancreas and the size of islets were measured using Fiji Is Just ImageJ (Schindelin *et al.* 2012). Islet numbers in each pancreas section were counted. In addition, the area of pancreas and the area of each islet were determined (*n* = 8–9/group). Average islet numbers per pancreas, average islet areas (mm^2^) per pancreas and the ratio (%) of islet area (mm^2^) to pancreas area (mm^2^) were calculated.

### Determination of plasma metabolic-related hormone levels

Plasma insulin, glucagon, total glucagon-like peptide 1 (Glp1), total peptide YY (Pyy) and leptin levels were determined by a mouse U-PLEX Diabetes Combo 1 kit using a Sector Imager 6000 instrument, according to the manufacturer’s instructions (Meso Scale Discovery, USA).

### Statistical analysis

The experimental data were analyzed using the GraphPad Prism 10, Version 10.4.0, for Windows 64-bit. Results are presented as the mean ± SEM. A one-way or two-way ANOVA test followed by a Tukey’s multiple comparisons test was used to calculate adjusted *P* values. An unpaired Student’s *t*-test was used to compare numerical data when appropriate. Differences were significant at *P* < 0.05.

## Results

### Male offspring born to the *Sst*KO dams were susceptible to diet-induced weight gain with extreme obesity

To investigate the influence of maternal *Sst* deficiency on an offspring’s risk of developing obesity, insulin resistance and type 2 diabetes in their adult life, we randomized both male and female *Sst*KO-MSD offspring born to the *Sst*-homozygous mothers and *Sst*KO and WT offspring born to the *Sst*-heterozygous mothers on a regular chow diet (CD) or a HFD with 45% kcal from fat (HFD) for 15 weeks from weaning. Figure 1A outlines the feeding and phenotyping experiments during the study. We observed that male *Sst*KO-MSD offspring maintained on CD had significantly reduced weight gain compared to the WT control by 17.4–18.8% starting from 6- to 7-weeks-old to the end of the study (18-week-old; *P* < 0.001). The average body weight of male *Sst*KO offspring fell between that of *Sst*KO-MSD and WT mice, but not significant to WT or to *Sst*KO-MSD (*P* > 0.05) (Fig. 1B). The growth of female *Sst*KO-MSD offspring displayed a similar trend when compared to the WT or *Sst*KO animals, but it was not significant (*P* > 0.05) (Fig. 1C). On CD, both male and female *Sst*KO-MSD mice tend to have less visceral fat than the *Sst*KO and WT counterparts (Fig. 1D and E). However, no statistical significance was detected for visceral fat or when the ratio of fat pad weight to body weight was calculated (Fig. 1F and G; *P* > 0.05).

Contrary to the male *Sst*KO-MSD offspring maintained on CD, the same genotypic male mice had significantly increased weight gain compared to the WT (7.6–17.1%; *P* < 0.01) or *Sst*KO mice (13.4–14.3%, *P* < 0.01) starting from weeks 11–12 after the introduction of HFD (Fig. 1B). On the other hand, with HFD, no difference was observed in weights between male *Sst*KO and WT mice (*P* > 0.05). Moreover, we observed that male *Sst*KO-MSD offspring challenged with HFD were severely obese compared to the male *Sst*KO or WT mice evidenced by a higher wet fat pad weight, including epididymal and retroperitoneal fat pads, and an increased ratio of fat pad weights to the final body weights at necropsy (Fig. 1F). We also demonstrated that the introduction of HFD in offspring increased weights and fat gains in all three genotypic groups of male mice compared to the respective mice fed CD (*P* < 0.05) (Fig. 1B and D). The impact of HFD was more significant in male *Sst*KO-MSD than the WT and *Sst*KO counterparts (Fig. 1B and D). The fat weight of the male *Sst*KO-MSD mice was increased by 3.8-fold (95 mg fat/g body weight) compared to the same genotypic mice fed CD (20 mg fat/g body weight; *P* < 0.001) (Fig. 1F). The increases in wet fat weights after HFD challenge were also observed in male *Sst*KO mice (34 mg fat/body weight) and the WT control (33 mg fat/body weight) compared to those fed CD (*Sst*KO, 24 mg/g body weight; WT, 26 mg/g body weight) (*P* < 0.05) (Fig. 1D). For female offspring, a significant difference in body weight gains between dietary groups was only observed in the *Sst*KO-MSD groups (*P* < 0.001). No significant difference between dietary groups was detected in the WT or *Sst*KO genotypic group after HFD challenge (*P* > 0.05) (Fig. 1E). Nonetheless, all female mice gained more fat after 15-week HFD challenge than those maintained on CD (*P* < 0.05) (Fig. 1E). However, only *Sst*KO-MSD mice displayed a significant increase of the ratio of fat pad weight to body weight (*P* < 0.05) (Fig. 1G). Taken together, male *Sst*KO-MSD mice, not the females, were highly susceptible to diet-induced obesity.

### The CD-fed *Sst*KO-MSD and *Sst*KO offspring had lower fasting and non-fasting blood glucose levels than the WT control

**Figure 2:**
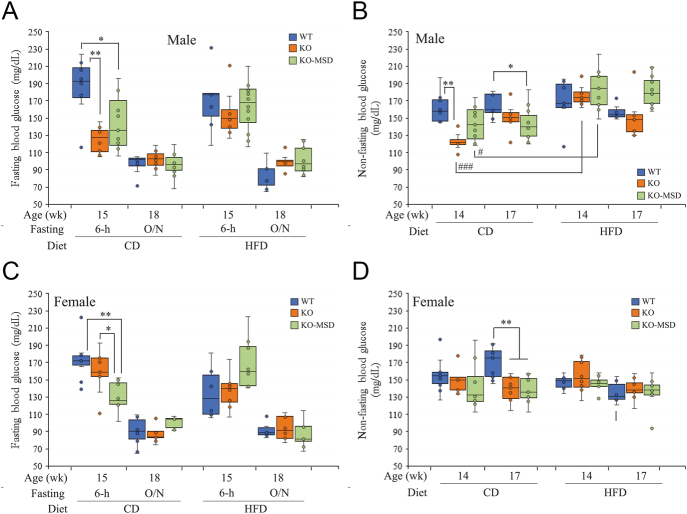
Fasting and non-fasting glucose levels. (A) Six-hour and overnight fasting glucose levels (mg/dL) after 12 and 15 weeks of dietary treatments (15 and 18 weeks of age) in male mice, respectively. (B) Non-fasting glucose levels (mg/dL) after 11 and 14 weeks of dietary treatment (14 and 17 weeks of age) in male mice, respectively. (C) Six-hour and overnight fasting glucose levels (mg/dL) after 12 and 15 weeks of dietary treatments (15 and 18 weeks of age) in female mice, respectively. (D) Non-fasting glucose levels (mg/dL) after 11 and 14 weeks of dietary treatment (15 and 18 weeks of age) in female mice, respectively. The data are shown as the mean ± SEM (*n* = 8–11/dietary group for males and *n* = 6–10/dietary group for females). *P < 0.05 and **P < 0.01 indicate the significant difference in genotypic groups in the same dietary group. ^#^*P* < 0.05 and ^###^*P* < 0.001 indicate the significant difference between diets in the same genotypic group. A one-way ANOVA test followed by a Tukey’s multiple comparisons test with adjusted *P* values was applied. WT, wildtype; KO, *Sst* knockout; KO-MSD, *Sst* knockout born to the *Sst*KO mothers; CD, chow diet; HFD, high-fat diet; wk, week. A full color version of this figure available at https://doi.org/10.1530/JME-24-0102.

Baseline blood glucose levels were measured at 15 weeks of age after 6 h fasting and at 18 weeks of age after overnight fasting in all offspring fed either CD or HFD. As shown in Fig. 2A, the average 6 h fasting blood glucose concentrations were lower in male *Sst*KO-MSD and *Sst*KO mice fed CD than the WT control (*Sst*KO-MSD, 145 mg/dL vs WT, 186 mg/dL, *P* < 0.05; *Sst*KO, 126 mg/dL vs WT, 186 mg/dL, *P* < 0.01). *Sst*KO mice had the lowest 6-h fasting blood glucose level among the three genotypic groups in the CD groups (*Sst*KO, 126 mg/dL vs *Sst*KO-MSD, 145 mg/dL, *P* < 0.01). However, no significant difference was observed in the overnight fasting blood glucose levels among these genotypic mice fed CD (*P* > 0.05). Meanwhile, the average non-fasting blood glucose levels measured in the morning hours at 08:00–09:00 h were also lower in male *Sst*KO-MSD and *Sst*KO mice than the WT control in the CD groups at 14 weeks of age (*Sst*KO, 120 mg/mL vs WT, 162 mg/dL, *P* < 0.01; *Sst*KO-MSD, 143 mg/mL vs WT, 162 mg/dL, *P* = 0.05) (Fig. 2B). At 17 weeks of age, the average non-fasting glucose level in the CD-fed male *Sst*KO-MSD remained lower than the WT control (*Sst*KO-MSD, 143 mg/mL vs WT, 164 mg/dL, *P* < 0.05), while no significant difference was detected between the CD-fed male *Sst*KO and WT mice (*Sst*KO, 156 mg/mL vs WT, 164 mg/dL, *P* > 0.05) (Fig. 2B). For female offspring maintained on CD, a similar trend of decreased blood glucose levels in the fasting and non-fasting conditions was observed in *Sst*KO-MSD compared to the WT mice at ages of 15 and 17 weeks, respectively (Fig. 2C and D).

### HFD challenge significantly increased non-fasting blood glucose levels in male *Sst*KO-MSD and *Sst*KO mice, but not the females

While HFD challenge has limited effect on fasting glucose levels (both 6 h and overnight) in male *Sst*KO-MSD and *Sst*KO mice (Fig. 2A), it significantly increased non-fasting blood glucose levels in these mice compared to their counterparts fed CD at 14-weeks-old by 44.1% (*P* < 0.001) and 21.5% (*P* < 0.05), respectively (Fig. 2B). These differences between dietary groups were not detected in male *Sst*KO-MSD and *Sst*KO mice at 17 weeks of age (Fig. 2B). Moreover, we did not notice any significant difference in both fasting and non-fasting blood glucose levels among the three genotypic male mice fed HFD (*P* > 0.05) (Fig. 2A and B). In the meantime, we did not observe differences in blood glucose levels in female mice between dietary groups (*P* > 0.05) nor among the genotypic groups challenged with HFD (Fig. 2C and D).

### Impaired insulin action in the male *Sst*KO-MSD offspring fed HFD

**Figure 3:**
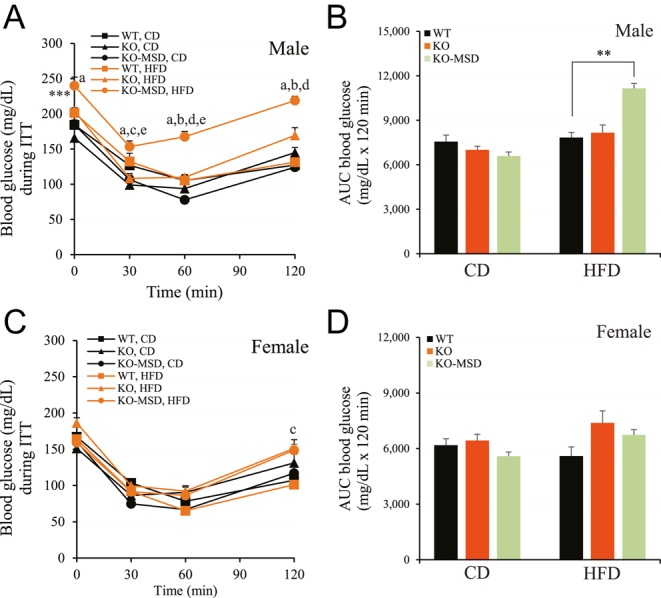
Insulin tolerance test of male and female mice at 16 weeks of age. (A) and (C) Blood glucose levels in male and female mice during ITT, respectively. (B) and (D) The area under the curve of blood glucose concentrations in male and female mice during ITT, respectively. The data are given as the mean ± SEM, *n* = 7–11/dietary group for males and *n* = 8–10/dietary group for females. **P < 0.01 indicates the significant difference between WT and *Sst*KO-MSD groups fed HFD. The letters indicate the significant difference (*P* < 0.05 or *P* < 0.01) between groups as follows: a = *Sst*KO-MSD mice between CD and HFD; b = *Sst*KO vs KO-MSD fed CD; c = WT vs *Sst*KO fed HFD; d = WT vs KO-MSD fed HFD; e = *Sst*KO vs KO-MSD fed HFD. A one-way ANOVA test (AUC glucose levels) or a two-way test (ITT) was used to compare the means of multiple groups followed by a Tukey’s multiple comparisons test. WT, wildtype; KO, *Sst* knockout; KO-MSD, *Sst* knockout born to the *Sst*KO mothers; CD, chow diet; HFD, high-fat diet; ITT, insulin tolerance test; AUC, area under the curve. A full color version of this figure available at https://doi.org/10.1530/JME-24-0102.

To assess the effect of maternal *Sst* deficiency on whole body sensitivity of insulin on adult offspring fed either CD or HFD, we performed insulin tolerant test (ITT) at 16 weeks of age, following 13 weeks of dietary treatment. As shown in Fig. 3A and C, both male and female *Sst*KO-MSD and *Sst*KO mice maintained on CD had no significant difference in blood glucose concentrations at the baseline and at the 120 min time point after the insulin administration compared to the WT control (*P* > 0.05). However, compared with the CD-fed WT mice, the CD-fed *Sst*KO-MSD mice had significantly enhanced insulin sensitivity, evidenced by the decreased blood glucose levels at the 30 min timepoint (females; *P* < 0.01) and the 60 min timepoint (male; *P* < 0.01) post-insulin challenge (Fig. 3A and C). Nonetheless, the area under the curves (AUC) calculated based on the glucose levels of each timepoint did not show significant difference among the three genotypic male or female mice fed CD (Fig. 3B and D).

Meanwhile, after HFD challenge, the enhanced insulin sensitivity observed in the CD-fed male *Sst*KO-MSD mice disappeared (Fig. 3A). Instead, these mice exhibited significant insulin resistance, with markedly elevated blood glucose levels at all timepoints examined during ITT (0, 30, 60 and 120 min) compared to the male *Sst*KO and WT mice in the same dietary group (*P* < 0.01) (Fig. 3A), which was also supported by the AUC of blood glucose concentrations during ITT (*P* < 0.01) (Fig. 3B). In females, after HFD challenge, only *Sst*KO mice exhibited slightly impaired insulin sensitivity at 120-min post-insulin injection when compared to the WT control (*P* < 0.05) (Fig. 3C). However, there was no difference in the AUC of blood glucose levels (*P* > 0.05) (Fig. 3D). Taken together, maternal *Sst* deficiency increases risk of diet-induced insulin resistance in male adult offspring.

### Reduced insulin secretion in the CD-fed *Sst*KO-MSD offspring during OGTT

**Figure 4:**
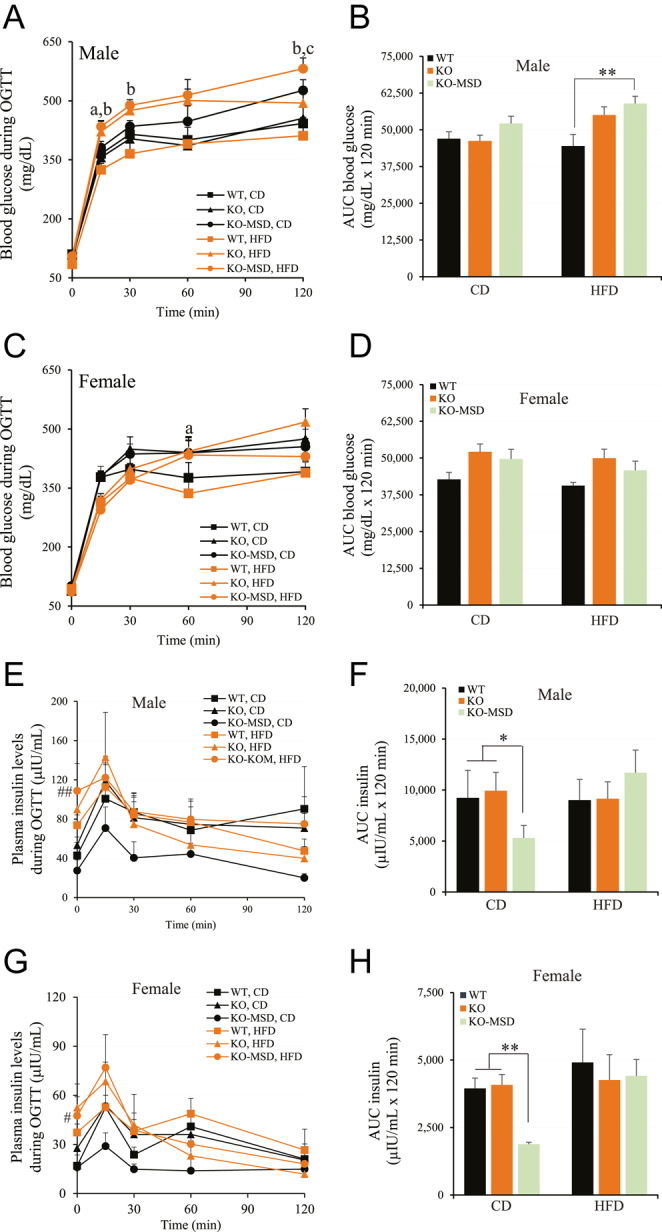
Oral glucose tolerance test at 18 weeks of age. (A) and (C) Blood glucose levels in male and female mice during OGTT, respectively. (B) and (D) The area under the curve of blood glucose concentrations in male and female mice during OGTT, respectively. (E) and (G) Plasma insulin levels in male and female mice during OGTT, respectively. (F) and (H) The area under the curve of plasma insulin concentrations in male and female mice during OGTT, respectively. The data are given as the mean ± SEM, *n* = 7–11/dietary group for males and *n* = 8–10/dietary group for females. *P < 0.05 and **P < 0.01 indicate the significant difference between *Sst*KO-MSD or *Sst*KO and WT mice within the same dietary group. The letters indicate the significant difference between groups as follows: a = WT vs *Sst*KO fed HFD (*P* < 0.05); b = WT vs *Sst*KO-MSD fed HFD (*P* < 0.01). c = WT vs *Sst*KO-MSD fed CD (*P* < 0.05). A one-way (AUC blood or insulin levels) or a two-way ANOVA test (blood glucose or insulin during OGTT) followed by a Tukey’s multiple comparisons test was used. WT, wildtype; KO, *Sst* knockout; KO-MSD, *Sst* knockout born to the *Sst*KO mothers; CD, chow diet; HFD, high-fat diet; OGTT, oral glucose tolerance test; AUC, area under the curve. A full color version of this figure available at https://doi.org/10.1530/JME-24-0102.

We further evaluated the body’s response to glucose in *Sst*KO-MSD, *Sst*KO and WT offspring by performing oral glucose tolerance tests (OGTT) at 18 weeks of age after 15 weeks on the treatment diets. As shown in Fig. 4A, in the HFD groups, male *Sst*KO-MSD or *Sst*KO mice displayed a profound glucose intolerance compared to the male WT mice, indicated by the significantly increased blood glucose at timepoints of 15-, 30- or 120-min post-glucose administration (*P* < 0.05). This observation was also reflected by the significantly increased AUC of glucose concentrations in *Sst*KO-MSD mice compared to the WT control (*P* < 0.01) during OGTT (Fig. 4B). Whereas there was no significant difference in the AUC of glucose among the three genotypic groups in the HFD-fed female mice (Fig. 4D), albeit *Sst*KO mice had higher blood glucose levels at 60 min post-glucose challenge during OGTT (*P* < 0.05) (Fig. 4C).

Contrary to the observation of a small, but significantly enhanced insulin sensitivity in the CD-fed male *Sst*KO-MSD mice at the 60-min timepoint during ITT (Fig. 3A), we noticed that these mice had slightly increased blood glucose at 120-min post-glucose administration during OGTT (*P* < 0.05) (Fig. 4A), hinting at a relative deficiency of insulin in male, but not female, *Sst*KO-MSD mice compared to the *Sst*KO and WT controls under the CD feeding regime.

We therefore quantitated insulin concentrations during OGTT for *Sst*KO-MSD, *Sst*KO and WT mice in both dietary groups. Consistently, both sexes of the CD-fed *Sst*KO-MSD mice had significantly lower insulin levels than *Sst*KO or WT mice in the same dietary group during OGTT, evidenced by a 42.5–52.4% reduction in the AUC of circulating insulin concentrations (*P* < 0.05, males; *P* < 0.01, females) (Fig. 4F and H). Nonetheless, we did not detect a noticeable difference in the circulating insulin levels during OGTT nor the AUC of insulin levels among the three genotypic mice fed HFD, including males and females (Fig. 4E, F, G, H).

### Reduced islet to pancreas area ratio in the CD-fed *Sst*KO-MSD mice

**Figure 5:**
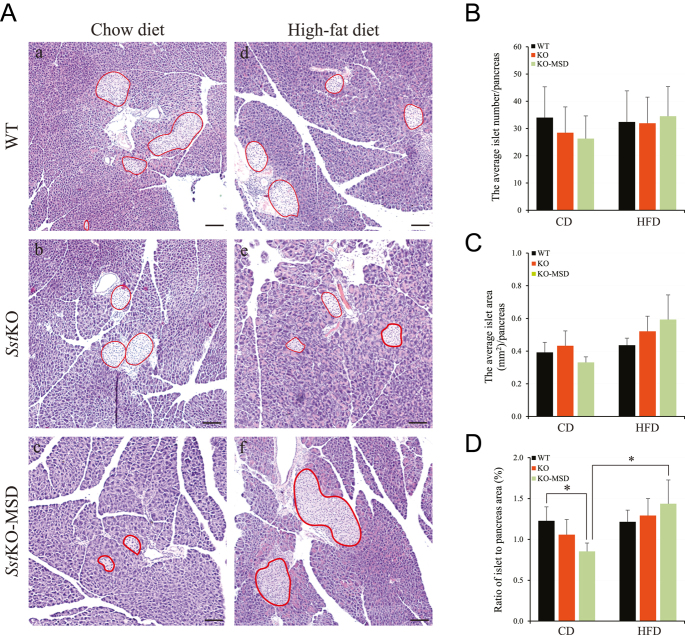
Pancreatic islet morphology and quantification of islet sizes and areas. (A) Representative microphotographs of the H&E-stained pancreases. The pale areas represent islet locations circled in red. Scale bar is 100 μm. (B) The average islet number per pancreas. (C) The average islet area (mm^2^) per pancreas. (D) Ratio of islet to pancreas area (%). All data are presented as the mean ± SEM, *n* = 8–11/group. *P < 0.05 indicates the significant difference between the specified groups. An unpaired Student *t*-test was used for analysis. CD, chow diet; HFD, high-fat diet; WT, wildtype; KO, *Sst* knockout; KO-MSD, *Sst* knockout born to the *Sst*KO mothers. A full color version of this figure available at https://doi.org/10.1530/JME-24-0102.

The impairment in the insulin secretion observed in the CD-fed *Sst*KO-MSD mice during OGTT prompted us to examine the integrity of pancreatic islets in these mice and compared it to that of *Sst*KO and WT controls. We quantified the islet numbers and areas and then quantified islet to pancreas area ratios using H&E-stained pancreas sections prepared from the experimental animals. As shown in Fig. 5A and D, the CD-fed *Sst*KO-MSD mice exhibited a significantly lower ratio of islet area to pancreas area than the WT control (*P* < 0.05). No significant difference was observed between *Sst*KO-MSD and *Sst*KO mice (*P* > 0.05). Likewise, when subjected to HFD, no significant differences were detected in islet numbers, islet areas and the ratio of islet area to pancreas area among the three genotypes (Fig. 5A, B, C, D). However, an increased average ratio of islet area to pancreas area was detected in *Sst*KO-MSD mice fed HFD compared to those maintained on CD, whereas this increase was not noticed in the *Sst*KO and WT control mice (Fig. 5D). Neither diet nor the genotype had a visibly negative impact on the morphological or histological characteristics of the pancreatic islets (Fig. 5A). Together, these results raise the possibility that an impaired islet development could be the underlying mechanism for the reduction in the glucose-stimulated insulin secretion observed in the *Sst*KO-MSD mice maintained on CD, while HFD challenge greatly promotes the islet expansion in these mice.

### Impact of maternal *Sst* deficiency on metabolic hormones

**Figure 6:**
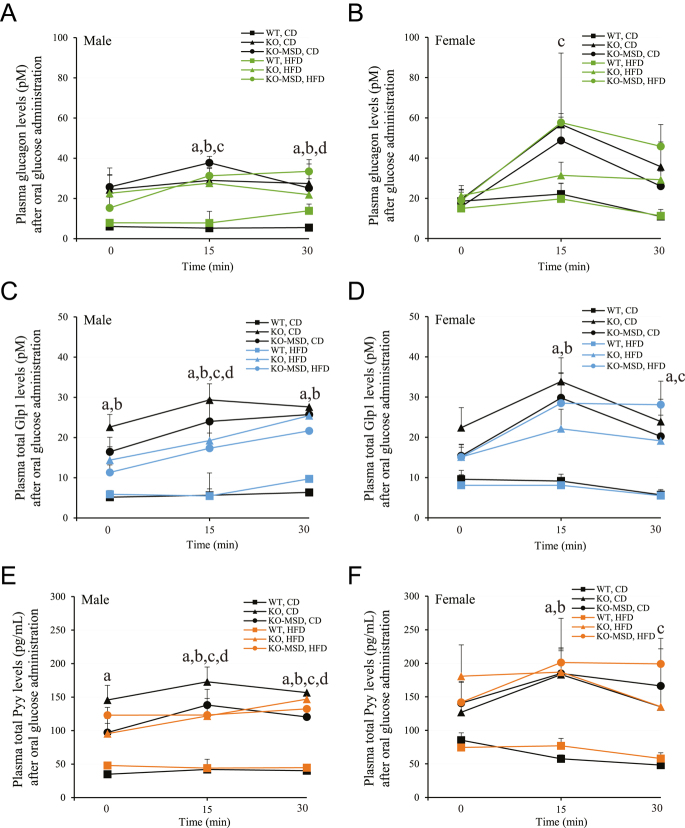
Plasma hormone levels after oral glucose stimulation in mice at 18 weeks of age. (A) and (B) Plasma glucagon levels in male and female mice, respectively. (C) and (D) Plasma total Glp1 levels in male and female mice, respectively. (E) and (F) Plasma total Pyy levels in male and female mice, respectively. The data are the mean ± SEM, *n* = 5–8/dietary group for males and *n* = 5–6/dietary group for females. The letters indicate the significant difference (*P* < 0.05 or *P* < 0.01) between groups as follows: a = WT vs *Sst*KO fed CD; b = WT vs *Sst*KO-MSD fed CD; c = WT vs *Sst*KO-MSD fed HFD; d = WT vs *Sst*KO fed HFD. A two-way ANOVA test followed by a Tukey’s multiple comparisons test was used. WT, wildtype; KO, *Sst* knockout; KO-MSD, *Sst* knockout born to the *Sst*KO mothers; CD, chow diet; HFD, high-fat diet. Glp1, glucagon like peptide 1; Pyy, peptide YY. A full color version of this figure available at https://doi.org/10.1530/JME-24-0102.

Given the potent inhibitory action of Sst on the endocrine system, we further examined the changes of several metabolic hormone levels in the experimental mice, including plasma glucagon-like peptide 1 (Glp1), peptide YY (Pyy) and glucagon (Gcg) using blood samples collected at 0-, 15- and 30-min timepoints during OGTT. Both male and female *Sst*KO-MSD and *Sst*KO mice had significantly higher plasma glucagon, Glp1 and Pyy levels throughout the 30-min course of OGTT, regardless of diets or sexes, when compared to the WT control (*P* < 0.05) (Fig. 6A, B, C, D, E, F). Moreover, diets and sexes had no significant effects on glucagon, Glp1 and Pyy secretion during the first 30 min of OGTT between *Sst*KO-MSD and *Sst*KO mice (*P* > 0.05). In the WT mice of both sexes, the secretion of Gcg, Glp1 and Pyy remained unchanged during OGTT between the two dietary treatments (Fig. 6).

### Significantly elevated plasma leptin concentrations in the male *Sst*KO-MSD mice challenged with HFD

**Figure 7:**
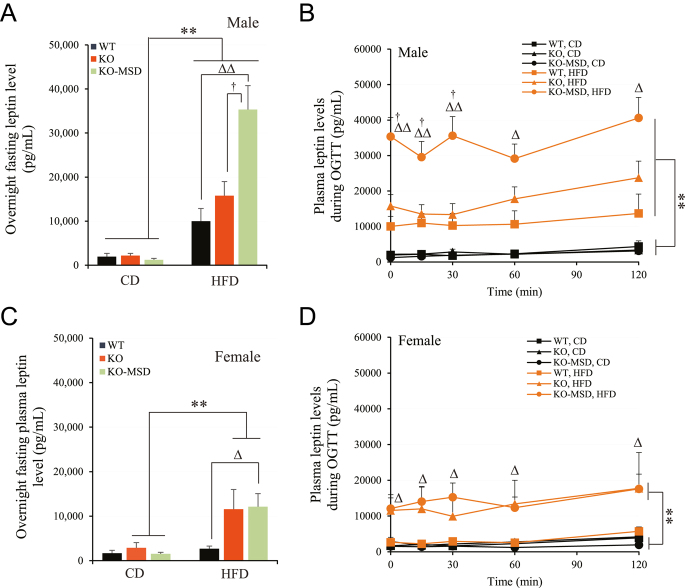
Plasma leptin levels in offspring during OGTT. (A) and (C) Overnight fasting plasma leptin levels in male and female offspring, respectively. (B) and (D) Plasma leptin levels during OGTT in male and female offspring, respectively. Data are the mean ± SEM; *n* = 7–8/male group and *n* = 5–6/female group. **P < 0.01 indicates the significant difference between diets in the same genotypic group. ^Δ^*P* < 0.05 and ^ΔΔ^*P* < 0.01 indicate the significant difference between *Sst*KO-MSD and WT mice fed HFD. ^†^*P* < 0.05 indicates the significant difference between *Sst*KO-MSD and *Sst*KO mice fed HFD. A two-way ANOVA test followed by a Tukey’s multiple comparisons test was used. WT, wildtype; KO, *Sst* knockout; KO-MSD, *Sst* knockout born to the *Sst*KO mothers; CD, chow diet; HFD, high-fat diet. A full color version of this figure available at https://doi.org/10.1530/JME-24-0102.

Leptin is synthesized and secreted from the adipose tissue. Given that male *Sst*KO-MSD mice challenged with HFD were susceptible to weight and fat gains (Fig. 1B, D, F), we investigated the plasma leptin levels of the experimental mice in the fasting condition and during OGTT. In the CD-fed mice, we did not detect noticeable changes in the overnight fasting plasma leptin concentrations in both male and female mice among the three genotypic groups (*P* > 0.05) (Fig. 7A). In contrast, HFD challenge greatly increased the fasting plasma leptin levels in *Sst*KO-MSD mice by 3.5-fold in males (*P* < 0.01) and by 4.5-fold in females (*P* < 0.01), compared to the WT mice in the same dietary group (Fig. 7A and C).

When comparing the genotypic mice between diets, the HFD-fed male *Sst*KO-MSD mice exhibited the greatest increase in the fasting plasma leptin levels (28.8-fold), followed by the male *Sst*KO mice (7.3-fold) and the male WT (5.1-fold) (*P* < 0.01) (Fig. 7A). A similar rise, but to a lesser extent in the fasting plasma leptin levels, was observed in the female *Sst*KO-MSD (3.7-fold) and *Sst*KO (4.0-fold) mice after HFD challenge, compared to the respective genotypic mice fed CD (Fig. 7C). The fasting plasma leptin levels remained comparable in the WT mice between the two dietary treatments in females (*P* > 0.05) (Fig. 7C). Oral glucose challenge had no apparent effects on plasma leptin levels in mice across the genotypic groups and between the diets when compared to the respective baselines during OGTT (Fig. 7B and D). Taking together, a lack of maternal Sst exposure conferred offspring susceptible to HFD-induced leptin resistance later in life.

### mRNA expression of genes associated with the leptin signaling pathway in the hypothalamus

**Figure 8:**
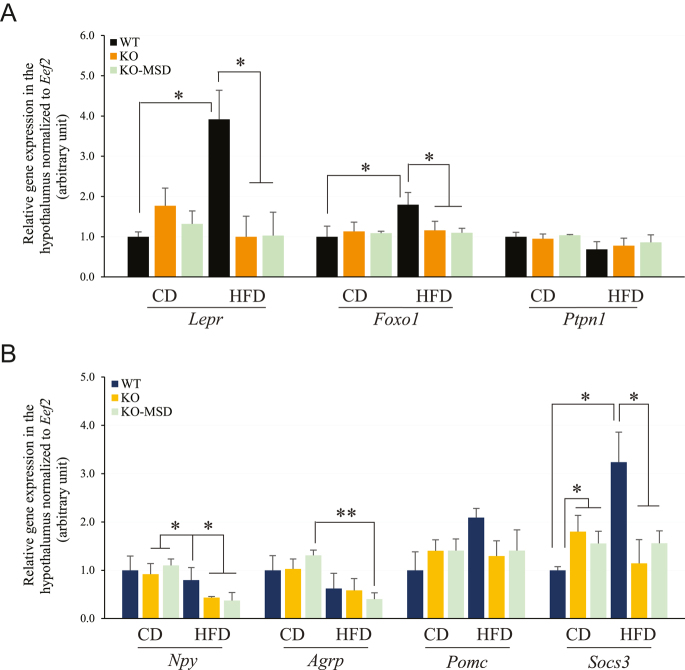
mRNA expression in the hypothalamus of male offspring. (A) Relative mRNA expression of *Lepr*, *FoxOl* and *Ptpn1*. (B) Relative mRNA expression of FoxO1 target genes in the hypothalamus. Total RNA was purified from the hypothalamic tissue in the brain and cDNA was then synthesized. The target gene expression was normalized to the expression of *Eef2*. Values are the mean ± SEM; *n* = 3. *P < 0.05 and **P < 0.01 indicate the significant difference between the specified groups. An unpaired Student *t*-test was used for analysis. WT, wildtype; KO, *Sst* knockout; KO-MSD, *Sst* knockout born to the *Sst*KO mothers; CD, chow diet; HFD, high-fat diet; *Lepr*, leptin receptor; *FoxO1*, forehead box O1; *Ptpn1*, protein tyrosine phosphatase non-receptor type 1; *Npy*, neuropeptide Y; *Agrp*, agouti related neuropeptide; *Pomc*, proopiomelanocortin; *Socs3*, suppressor of cytokine signaling 3; *Eef2*, eukaryotic translation elongation factor 2. A full color version of this figure available at https://doi.org/10.1530/JME-24-0102.

In the hypothalamus, leptin binds to the leptin receptor (Lepr) in the arcuate nucleus, stimulating the downstream leptin signaling pathway and subsequently affecting Npy/Agrp neurons, leading to an inhibition of food intake by competing for the function of FoxO1 (forkhead box protein O1) (Kitamura *et al.* 2006). The effective genes of the leptin signaling or the target genes of FoxO1 transcription factor include *Lepr*, *FoxO1*, *Ptpn1* (protein tyrosine phosphatase non-receptor type 1, also called protein-tyrosine phosphatase 1B, Ptp1b), *Npy* (neuropeptide Y), *Agrp* (agouti-related peptide), *Pomc* (proopiomelanocortin) and *Socs3* (suppressor of cytokine signaling). FoxO1 is expressed in the key leptin target neurons in the hypothalamus, and its activation is induced by fasting to increase food intake (Kim *et al.* 2006). Ptpn1 is a ubiquitously expressed negative regulator of Lepr-mediated signaling and has subsequent effects on energy balance (Frangioni *et al.* 1992). *Npy*, *Agrp*, *Pomc* and *Socs3* are the transcriptional target genes of FoxO1, and their expression or suppression regulates food intake. We therefore examined mRNA expression of these genes in the male hypothalamus. The results showed that HFD challenge significantly stimulated *Lepr* mRNA expression (3.9-fold) in the WT hypothalamus compared to that in the CD-fed WT ones (Fig. 8A and Supplementary Fig. 1 (see section on Supplementary materials given at the end of the article)). However, this HFD-induced increase in *Lepr* gene expression was blunted in the male *Sst*KO-MSD hypothalamus. *Lepr* expression was also suppressed in the male *Sst*KO hypothalamus after HFD challenge. A similar result was found for *FoxO1*, *Npy* and *Socs3* in the HFD-fed hypothalamus (Fig. 8B and Supplementary Fig. 1). However, the mRNA expression of *Ptpn1* and *Pomc* was not affected by the dietary treatments or by the genotypes (Fig. 8A and B). It is worth noting that the *Agrp* expression tended to be downregulated in all three genotypic mice after HFD challenge, compared to CD, but only this aspect in *Sst*KO-MSD mice reached significance (*P* < 0.001) (Fig. 8B). Furthermore, we noted that *Socs3* was the only one among the examined genes that exhibited increased gene expression in the CD-fed *Sst*KO-MSD and *Sst*KO hypothalamus compared to the WT control (*P* < 0.05) (Fig. 8B). Together, the results suggest that HFD challenges upregulated mRNA expression of genes involved in downregulating food intake in the hypothalamus. However, this regulatory mechanism was disrupted in both *Sst*KO-MSD and *Sst*KO mice.

## Discussion

It has been well accepted that alterations in the maternal environment during pregnancy can have long-term metabolic consequences in offspring, including susceptibility to diet-induced obesity (Nilsson *et al.* 2001, Krechowec *et al.* 2006, Alejandro *et al.* 2014, Wu *et al.* 2023). The intrauterine environment, in particular maternal genotypes, interacts with fetal genomics to determine health and disease status in adult offspring (Yamashita *et al.* 2003, Burchat *et al.* 2021). Somatostatin, an inhibitory peptide for many metabolic hormones involved in regulating glucose and energy metabolism, plays a critical role in fetal growth and development (Goldstein *et al.* 1995). However, the health consequences, such as the susceptibility to diet-induced obesity and type 2 diabetes in adult offspring caused by a complete lack of *Sst* expression from both the dam and the fetus during pregnancy, were largely unknown. The current study aimed to investigate the impact of maternal *Sst* genotypes on glucose and insulin metabolism of adult offspring maintained on CD or HFD with 45% kcal from fat for 15 weeks from weaning. To achieve this, we conducted a series of phenotyping studies in the adult offspring born to the dams with either the heterozygous or homozygous *Sst* genotype after the specific diet was introduced. We discovered that a complete absence of maternal Sst exposure led to an increased risk of diet-induced weight gain, obesity and insulin resistance in male adult offspring. Impairment of pancreatic islet development was evident when *Sst*KO-MSD mice were maintained on CD. Metabolic hormones, such as glucagon, Glp1 and Pyy, were significantly increased in *Sst* knockout mice, including *Sst*KO-MSD and *Sst*KO genotypes. Moreover, male *Sst*KO-MSD offspring displayed severe leptin resistance accompanied by blunted gene expression of *Lepr*, *FoxO1*, *Agrp*, *Npy *and *Socs3* in the hypothalamus after HFD challenge. The HFD-induced weight gains and obesity in male *Sst*KO and WT mice born to the heterozygous dams were relatively mild, with no significant difference observed between the two groups. This result is consistent with a previous study by Luque *et al.* (2016*b*), where male *Sst*KO and WT mice of the same age (17–18 weeks) showed similar body weights and no obvious diet-induced obesity after HFD challenge, compared to the mice fed a low-fat control diet.

In the current study, we demonstrated that both male and female mice exhibited a tendency of HFD-induced body weight gains, regardless of genotypes. However, females showed much smaller HFD-induced weight gains and fat accumulation than males. This phenomenon was also reported by Casimiro *et al.* and Kępczyńska *et al.*, in which the authors concluded that females had less pronounced phenotypic changes in response to the HFD challenge (Casimiro *et al.* 2021, Kępczyńska *et al.* 2021). One potential explanation for this difference could be the protective effects of estrogen, which has been implicated in the regulation of energy balance through mechanisms, such as enhancing lipid oxidation, reducing food intake and promoting energy expenditure via estrogen receptor α (ERα) activation in the hypothalamus (Clegg *et al.* 2006). Differences in fat distribution may further contribute to these sex-specific outcomes. Females are more likely to store fat subcutaneously that tends to be less metabolically active via the action of estrogen (Tchernof & Despres 2013), while males tend to accumulate visceral fat (Vague 1956, Clegg *et al.* 2006). Visceral fat is metabolically active and prone to inflammation, which is a factor determining insulin sensitivity and responding to metabolic challenges (Vague 1956). Moreover, the lack of pronounced phenotypes in females may be partly influenced by their more continuous GH secretion pattern that differs from the more pulsatile pattern observed in males. The pattern of continuous GH secretion in females could confer a relative protection against GH resistance induced by *Sst* knockout (Ho *et al.* 1988, Veldhuis *et al.* 2006). Thus, the results from the current study and others emphasized the importance of considering sex differences in the context of obesity pathophysiology and treatments.

The impact of intrauterine *Sst* deficiency on the risk of diet-induced obesity in adult offspring has not been completely known, although other intrauterine factors are known to increase the susceptibility of adult offspring to metabolic diseases, such as the presence of maternal obesity or diabetes. When mice are exposed to such intrauterine influences, their vulnerability to diet-induced obesity intensifies (Yamashita *et al.* 2003, Tamashiro *et al.* 2009), leading to further detrimental metabolic consequences including cardiovascular diseases (Samuelsson *et al.* 2008). Factors that contribute to the development of offspring adiposity include i) persistent alterations in hypothalamic centers, which regulates energy balance and locomotor behavior (Samuelsson *et al.* 2008); ii) activation of hepatic lipogenesis coupled with impaired beta-oxidation (Ornellas *et al.* 2015); and iii) disrupted adipocyte metabolism characterized by increased adipogenesis and reduced lipolysis (Samuelsson *et al.* 2008). All of these alterations further cause hypothalamic leptin resistance (Ferezou-Viala *et al.* 2007) and insulin resistance (Samuelsson *et al.* 2008, Nivoit *et al.* 2009), conditions frequently observed in obese offspring. Sst plays a central role in the hormonal network that regulates glucose and insulin metabolism and food intake. This intricate network, which encompasses various hormones, collaboratively works to maintain proper glucose levels within the body in either fasting or postprandial status (Reichlin 1983*a*,*b*, Adriaenssens *et al.* 2015). We demonstrated that dams with the *Sst* null genotype resulted in a higher propensity toward offspring developing diet-induced obesity and insulin resistance and leptin resistance later in life. Hence, the metabolic abnormalities observed in *Sst*KO-MSD mice are reminiscent of those associated with maternal gestational obesity or diabetes (Kweon *et al.* 2023).

Male *Sst*KO-MSD and *Sst*KO offspring shared the same genetic deficiency of *Sst*. However, *Sst*KO-MSD mice endured a complete *Sst* deficiency during fetus development. Male *Sst*KO-MSD mice, when compared to the *Sst*KO mice born to the heterozygous mothers, showed worsened glucose intolerance without dietary challenge, albeit these mice had improved insulin sensitivity evidenced in the insulin tolerance test. This phenotype could be attributed to the reduced ratio of islet area to pancreas area and reduced insulin production when *Sst*KO-MSD was maintained on CD. This likely compromised the ability to maintain euglycemia in these mice. The slightly elevated insulin sensitivity observed in the CD-fed *Sst*KO-MSD mice might be a compensatory response to the impaired islet development. Hence, this suggests that pancreatic islet development is Sst-dependent during the growth of mouse fetus.

Under HFD, both male *Sst*KO-MSD and *Sst*KO mice develop glucose intolerance compared to the WT control. In male *Sst*KO mice, insulin sensitivity and circulating insulin levels were comparable to the WT control during OGTT. Therefore, glucose intolerance observed in male *Sst*KO mice was likely caused by an increased fat deposit and a possible impaired hormone network due to the absence of somatostatin, which negatively regulates multiple hormone secretions, including insulin, glucagon, Glp1 and Pyy, and GH (Vu *et al.* 2001, Molitch 2011, Adriaenssens *et al.* 2015, Sekiya *et al.* 2020). Notably, the HFD-fed male *Sst*KO-MSD mice presented a significant increase in insulin levels during OGTT, accompanied by an increase in the ratio of islet area to pancreas area when compared to that of the CD-fed *Sst*KO-MSD mice. HFD is known to trigger β-cell mass expansion through enhanced β-cell proliferation, a response that can either precede or follow the onset of insulin resistance (Weir & Bonner-Weir 2004), resulting in a compensatory increase in insulin secretion to correct hyperglycemia. Thus, it is plausible that dietary fat intake was able to stimulate islet mass expansion and improved insulin secretion from β-cells in male *Sst*KO-MSD mice, albeit a relative normal euglycemia was not maintained compared to the WT control, which may be likely due to severe leptin resistance observed in these mice.

It is well-known that somatostatin inhibits GH secretion, which subsequently suppresses the activity of the GH-IGF1 (insulin-like growth factor 1) axis (Orskov 1996). Sst deficiency can increase the baseline GH secretion in mice due to the absence of Sst’s inhibitory effect on GH secretion. It has been shown that chronic elevations of GH levels in the circulation may lead to peripheral GH resistance, which is associated with increased adiposity and insulin resistance via the GH-IGF1 axis (Orskov 1996, Pedraza-Arevalo *et al.* 2015). Thus, we hypothesize that this mechanism may play a role in the phenotypes observed in *Sst*-KO-MSD and *Sst*-KO mice, particularly in the context of diet-induced obesity. Future studies could address this by measuring circulating GH, IGF1 levels and signaling markers of GH resistance, such as the Janus kinases (JAKs)-STAT signaling pathway, in the liver and adipose tissue to further elucidate the role of this axis in the observed metabolic outcomes.

Chronic HFD exposure is known to trigger low-grade systemic inflammation, primarily through macrophage infiltration in adipose tissue, and increased secretion of pro-inflammatory cytokines, such as TNF-α and IL-6 (Gregor & Hotamisligil 2011). These inflammatory mediators impair insulin signaling by disrupting key signaling pathways, including the phosphorylation of insulin receptor substrate (IRS) proteins and activation of stress kinases (Shoelson *et al.* 2007, Gregor & Hotamisligil 2011). The resulting insulin resistance can further exacerbate metabolic complications and promote adiposity. Previous studies by others have shown that somatostatin plays an important role in negating the chronic inflammation induced by excess body fat accumulation. It primarily acts via downregulating food intake and/or upregulating energy expenditure through hypothalamic neurons in the central nervous system, resulting in a decrease in obesity-induced inflammation (Kumar & Singh 2020, Massa *et al.* 2023). We showed that the mRNA expression of *Socs3* decreased in the hypothalamus of *Sst*KO-MSD and *Sst*KO mice. Socs3 is a suppressor of cytokine signaling 3 that controls inflammatory responses via JAKs/STAT3 signaling pathway and other cytokine-mediated signaling pathways, such as IL2, IL-6, IL12 and IFNs (Carow & Rottenberg 2014). Our study suggests that inflammatory signaling pathways may likely be less inhibited due to *Sst* knockout in mice. Hence, chronic inflammation may also contribute to the observed metabolic phenotypes in *Sst*-KO-MSD or *Sst*-KO mice in this study. Future studies are needed to illustrate the effects of the functional interaction between Sst and Socs on the activity of the inflammatory signaling pathways in *Sst*KO-MSD mice and compared to that in *Sst*KO mice, which would provide evidence for the importance of Sst exposure in the uterus during fetal development for reducing risks of metabolic diseases in adulthood.

Leptin resistance, mainly caused by the decrease in tissue sensitivity to the circulating leptin, results in hyperleptinemia, hyperphagia, obesity and metabolic disorders (Gruzdeva *et al.* 2019). The mechanisms underlying the development of leptin resistance include a decrease in the leptin signaling in the hypothalamus of the brain due to genetic defects in genes, including leptin, leptin receptors or others associated with leptin production, and transporting across the blood–brain barrier, and proteins involved in the leptin signaling pathway (Gruzdeva *et al.* 2019). The binding of leptin to the leptin receptor triggers the recruitment and autophosphorylation of JAK2, which subsequently activates the PI3K/PDK1/FoxO1 signaling pathway in the neuronal populations of Npy, Agrp, Socs3 and Pomc in the hypothalamus (Zhou & Rui 2013). FoxO1 is inactivated by the leptin signal in a PI3K-dependent manner (Park & Ahima 2014), which removes the transcriptional suppression of FoxO1 on *Socs3* and *Pomc*, reducing *Npy* and *Agrp* transcription in the hypothalamic neurons resulting in reduced body weight gain (Iskandar *et al.* 2010). In the current study, we observed a compensatory enhancement of *Lepr*, *FoxO1* and *Socs3* expression in the hypothalamus from the WT animals challenged with HFD. These observations are supported by previous studies suggesting that high-fat feeding in rodents can induce the expression of *Socs3* and resistance to leptin associated Stat3 activation in Pomc-, Npy- and Agrp-expressing neurons (Sainz *et al.* 2015). On the other hand, we did not observe the upregulation of transcriptional changes of *Lepr*, *FoxO1* and *Socs3* in both *Sst*KO-MSD and *Sst*KO mice challenged with HFD, suggesting the presence of leptin resistance in these mice.

We show in this study that a major difference between *Sst*KO-MSD and *Sst*KO was that *Sst*KO-MSD suffered an impaired islet development accompanied by reduced insulin secretion when maintained on CD, which was likely caused by the lack of maternal somatostatin exposure. However, we demonstrate that the islet expansion and insulin secretion in response to high-fat intake in the *Sst*KO-MSD mice were comparable to the WT control and to the *Sst*KO mice, indicating that the function of β-cells to synthesize enough insulin to maintain normoglycemia remained relatively intact in *Sst*KO-MSD mice.

We present that the average plasma leptin level in male *Sst*KO-MSD offspring was about two times greater than that in male *Sst*KO mice during fasting state and during OGTT after HFD challenge. This phenomena could be explained by i) increased adiposity in the male *Sst*KO-MSD offspring as leptin secretion is in proportion to the degree of obesity (Chessler *et al.* 1998) and ii) decreased insulin sensitivity in the male *Sst*KO-MSD offspring as plasma leptin levels are often inversely correlated with insulin sensitivity (Echwald *et al.* 1999, Hung *et al.* 2006, Dagogo-Jack 2024). In humans, healthy individuals with higher circulating leptin levels are often accompanied by lower insulin sensitivity independent of adiposity, suggesting that hyperleptinemia could be a compensatory response to insulin resistance (Chiriaco *et al.* 2024), which might lead to more adverse effects on metabolic outcomes (Zhao *et al.* 2019). In fact, blocking compensatory leptin response to insulin resistance by inhibiting its synthesis or its effector actions has been implied to be a potential mean to improve metabolic phenotype imposed by insulin resistance (Zhao *et al.* 2019). Furthermore, the association of insulin with leptin is bidirectional and is a part of the complex feedback of the adipoinsular axis that regulates metabolic responses between adipose tissue and pancreatic beta cells (Saad *et al.* 1998). Since plasma leptin levels in the male *Sst*KO-MSD offspring were increased so dramatically while glucose-stimulated insulin secretion was similar to the *Sst*KO and WT controls after HFD challenge, we speculate that hyperleptinemia observed in the male *Sst*KO-MSD mice is unlikely induced by insulin levels in the circulation. We therefore cannot exclude the condition that *Sst* deficiency in the uterine negatively affects the insulin signaling pathway in the insulin-responsive tissues in addition to affecting islet development in the pancreas.

In conclusion, our study sheds light on the significant role of maternal somatostatin expression during fetal development in influencing the metabolic outcomes of adult offspring, particularly in the context of diet-induced obesity. We found that a complete absence of maternal *Sst* expression increased the risk of male offspring to diet-induced obesity and severe insulin and leptin resistance. These findings emphasize the importance of the intrauterine environment in determining metabolic health of offspring later in life. In addition, our study uncovered the potential mechanisms underlying these metabolic changes, including alterations in pancreatic islet development, insulin sensitivity, leptin resistance and mRNA expression profiles of the genes involved in the leptin signaling pathway. These findings contribute to our understanding of the intricate hormonal network governing glucose and insulin metabolism and highlight the need for further investigation into the comprehensive signaling pathways affected by maternal somatostatin deficiency.

## Supplementary materials



## Declaration of interest

The authors declare that there is no conflict of interest that could be perceived as prejudicing the impartiality of the work reported.

## Funding

This work was supported by USDA/ARS/Western Human Nutrition Research Center project funds (2032-51000-005-00D) and the Henry A Jastro Scholarship awarded to Zhongyue Yang from the College of Agricultural and Environmental Sciences at University of California at Davis. USDA is an equal opportunity provider and employer. The content is solely the responsibility of the authors and does not necessarily represent the official views of the USDA.

## Author contribution statement

LH planned and designed work for this study. She established the mouse lines used in this study. She also analyzed the data, participated in drafting the manuscript and finally approved the manuscript. ZY maintained mouse lines, bred mice for experiments and performed dietary challenges in the experimental mice. She also performed phenotyping study and drafted the manuscript. In addition, ZY carried out MSD work for determination of plasma hormone levels. CPK performed immunohistochemistry. She also participated in OGTT and ITT studies and edited the manuscript.

## Data availability

Datasets generated during the current study are available from Dr Liping Huang (USDA/ARS/Western Human Nutrition Research Center, liping.huang@usda.gov) upon reasonable request.

## Ethics approval

All animal experiments were conducted in accordance with the National Institutes of Health Guidelines for the Care and Use of Experimental Animals and animal protocols were approved by the Institutional Animal Care and Use Committee of the University of California, Davis.
