# Supplementary material for: Lack of maternal exposure to somatostatin leads to diet-induced insulin and leptin resistance in mouse male offspring
Source: J Mol Endocrinol. 2025 Mar 22;74(4):e240102. doi: 10.1530/JME-24-0102 (PMC11964479; doi:10.1530/JME-24-0102)
Supplement: Supplementary file 1 [file supplementary_materials.pdf]

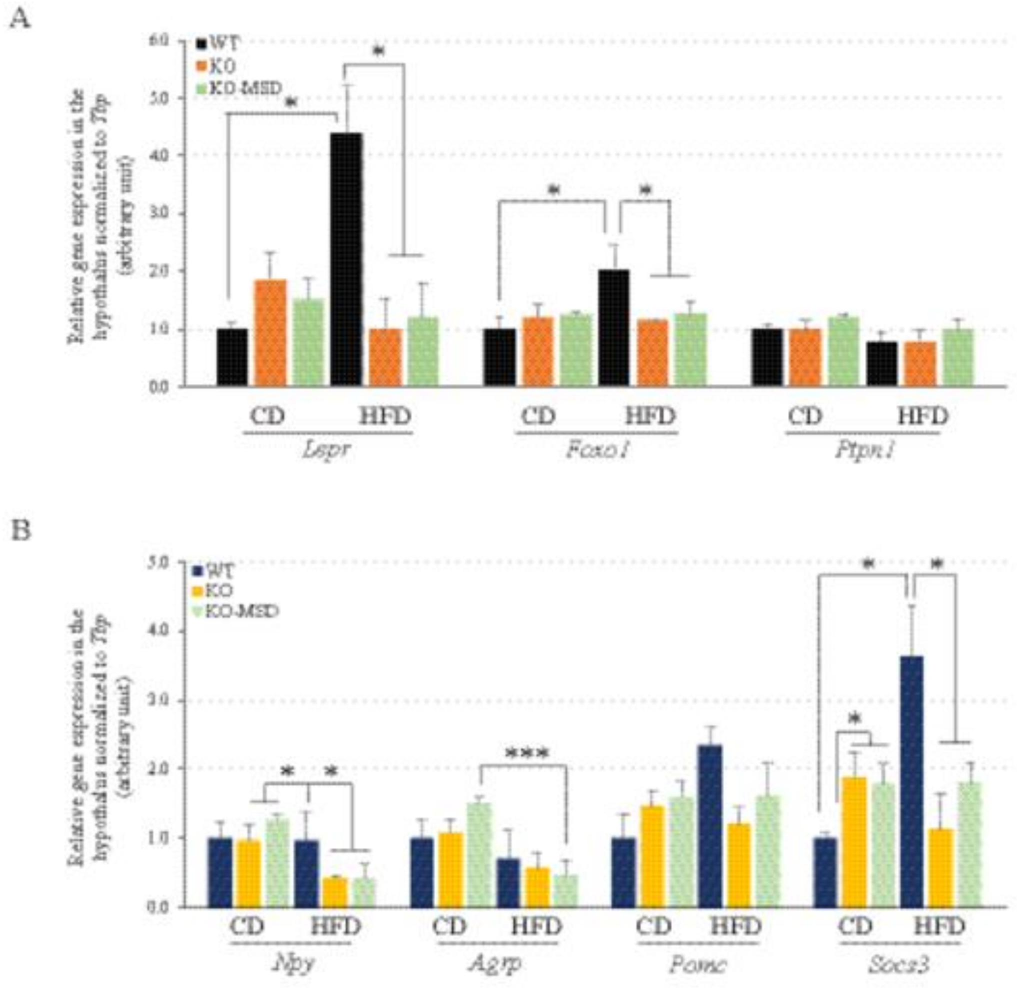

Suppl. Fig. 1. mRNA expression in the hypothalamus of male offspring. A. Relative mRNA expression of *Lepr*, *Foxol*, and *Ptpn1*. B. Relative mRNA expression of Foxo1 target genes in the hypothalamus. Total RNA was purified from the hypothalamic tissue in the brain and cDNA was then synthesized. The target gene expression was normalized to the expression of *Tbp*. Values are Mean ± SEM; n = 3. *, $p < 0.05$ and ***, $p < 0.001$ indicates the significant difference between the specified groups. An unpaired and parametric Student *t*-test was used for analysis. WT, wild type; KO, *Sst* knockout; KO-MSD, *Sst* knockout born to the *Sst*KO mothers; CD, chow diet; HFD, high-fat diet; *Lepr*, leptin receptor; *Foxo1*, forehead box O1; *Ptpn1*, protein tyrosine phosphatase non-receptor type 1; *Npy*, neuropeptide Y; *Agrp*, agouti related neuropeptide; *Pomc*, proopiomelanocortin; *Socs3*, suppressor of cytokine signaling 3; *Tbp*, TATA-box binding protein.
